# First case of *Chlorella* wound infection in a human in Australia

**DOI:** 10.1002/nmi2.50

**Published:** 2014-05-27

**Authors:** J Hart, L Mooney, I Arthur, T J J Inglis, R Murray

**Affiliations:** 1 Division of Microbiology and Infectious Diseases, PathWest Laboratory Medicine WA, Queen Elizabeth II Medical CentreNedlands, Australia; 2 Department of Orthopaedics, Bunbury Regional HospitalBunbury, Australia; 3 School of Pathology and Laboratory Medicine, Faculty of Medicine, Dentistry and Health Sciences, University of Western AustraliaCrawley, Australia

**Keywords:** algae, Australia, *Chlorella*, water-borne infection

## Abstract

A 30-year-old man developed an infected knee wound 2 days after jumping his bicycle into a freshwater dam. He required repeated debridement and tissue grew bright green colonies typical of the alga *Chlorella* plus *Aeromonas hydrophila*. This, and one previously reported case, responded to surgical debridement and careful wound management.

## Case

**Figure 1:**
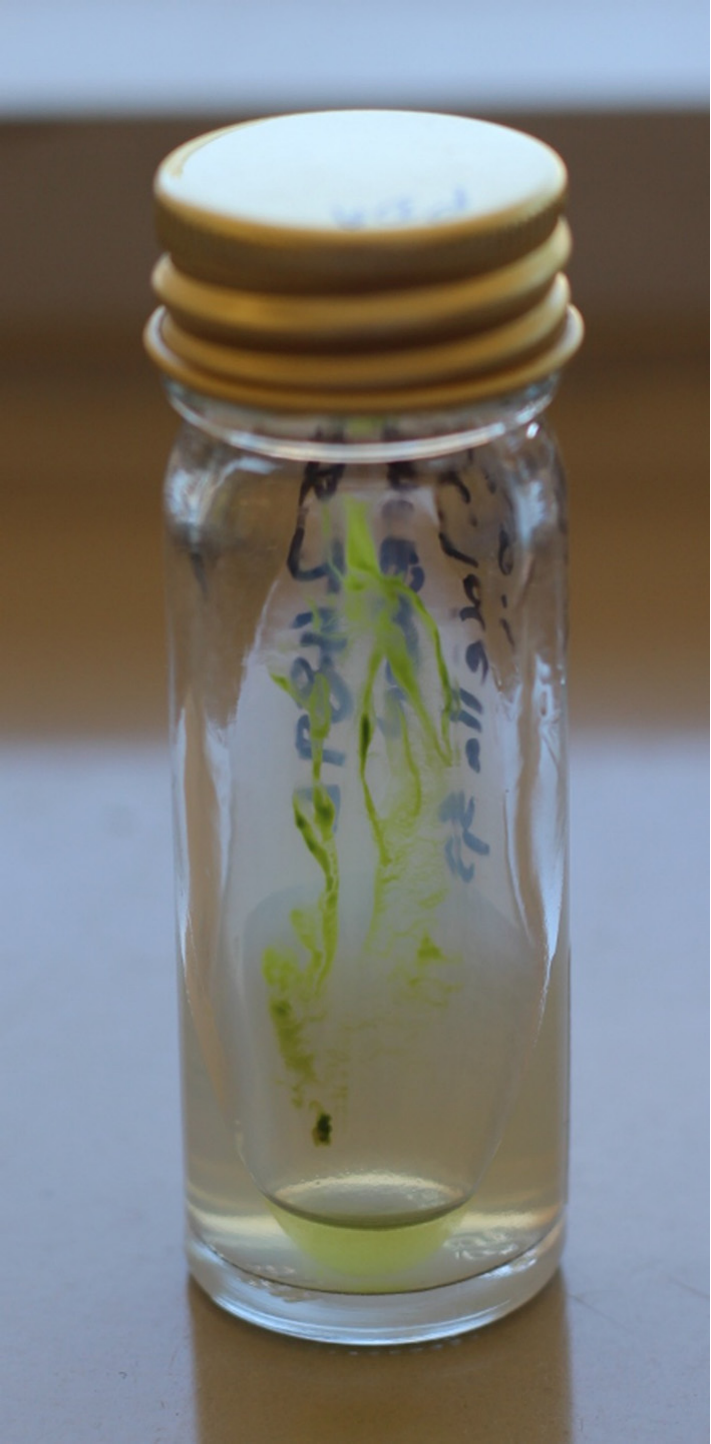
The bright green colonies of *Chlorella* growing on malt agar slope.

**Figure 2:**
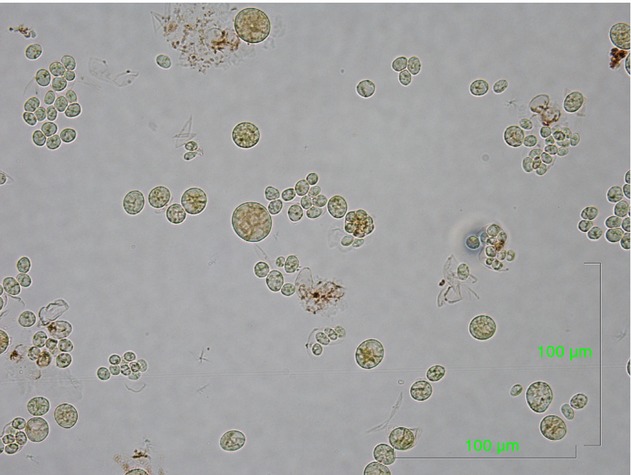
Wet mount microscopy (magnification ×1000) demonstrating round to oval 4–5 μm diameter cells with internal septation and a bright green colour, consistent with the alga *Chlorella*.

A 30-year-old man attended a rural hospital in Western Australia after lacerating the anterior aspect of his knee jumping his bicycle into a freshwater dam. The wound was cleaned and sutured, but 2 days later he developed serous discharge, with erythema and oedema to the surrounding tissue. His temperature was 38.0°C and his knee range of motion was limited, but not suggestive of a septic arthritis. Investigations revealed a C-reactive protein of 141 mg/L and leucocyte count of 7.0 × 10^9^/L. Plain X-ray demonstrated pre-patellar swelling, but no fracture or intra-articular effusion. At operation there was green-brown pus over the fat and bursal tissues. The infection had aggressive destructive features with necrotic fat and extension into the paratenon of the patellar tendon requiring debridement and pre-patellar bursectomy. Copious lavage of the wound was followed by application of a negative pressure wound dressing and commencement of empiric intravenous piperacillin/tazobactam. On day 3 of admission the wound debridement and negative pressure dressing application were repeated, with significant granulation tissue formation. Deep tissue specimens grew *Aeromonas hydrophila* and he was commenced on oral ciprofloxacin. After 2 weeks, bright green colonies grew on malt agar incubated at 37°C (Fig.1) and Sabouraud dextrose agar (with chloramphenicol) incubated at 26°C. Microscopy by wet mount (Fig.2) demonstrated bright green unicellular spherical 4–5 μm diameter cells with internal septation, consistent with the algae *Chlorella* spp. The wound had healed by secondary intention by the third week of negative pressure dressings and no further complication was noted.

## Chlorella

*Chlorella* spp. are unicellular algae of the Family Chlorellaceae and are found in fresh water, salt water and soil. *Chlorella* are distinguished from the closely related, more frequently reported, opportunistic alga *Prototheca* by the presence of chloroplasts producing a bright green colour and strongly periodic acid–Schiff-positive starch granules 1. Because they are rich in chlorophyll, *Chlorella* spp. are used diversely, for example as a nutritional supplement, in wastewater purification and in biodiesel production. Reproduction is similar to Prototheca, via endosporulation, an asexual process of cytoplasmic division by internal septation whereby the parent cell (sporangia) produces 2–20 endospores that enlarge in size to break from the sporangia 1.

Chlorellosis has been reported in limited numbers in sheep and cattle, and single cases in a human, gazelle, dog, beaver, camel and fish 2–8. Animals appear to be infected by exposure to contaminated water and disease ranges from localized cutaneous and lymph node infection to multi-organ dissemination 2. The only previously reported human case 3 was a 30-year-old female who underwent removal of a traumatic neuroma on the right foot and subsequent tenolysis, and then presented with persistent infection in the surgical wound 1 month after canoeing in a river in Nebraska. She was treated with debridement, chlorine solution whirlpool treatments, Dakin's solution irrigation and gauze packing, with eventual healing of the wound after 10 months. The debrided tissue was processed only for histopathology and was stained by periodic acid–Schiff, Grocott's methenamine silver and Gridley fungal stains, demonstrating round to oval organisms averaging 9 μm in diameter, containing large multiple granules in the cytoplasm. Electron microscopy showed degenerate organisms with structures suggestive of chloroplasts.

## Conclusion

*Chlorella* infection should be considered in wounds contaminated with fresh water from rivers and dams. Both human cases appear to have responded to surgical debridement and wound management. These strategies should be the main focus of freshwater-contaminated wound management, to prevent and treat infections with unusual environmental organisms, such as *Chlorella*.
